# The predictive value of the metabolic score for insulin resistance for metabolism-related disorders and fertility outcomes in Chinese women with polycystic ovary syndrome

**DOI:** 10.3389/fendo.2025.1716287

**Published:** 2025-12-05

**Authors:** Fengjuan Lu, Hong Yu, Baichao Shi, Jiaxing Feng, Yang Liu, Muxin Guan, Jiannan Yu, Zhuwei Gao, Jingshu Gao, Hongli Ma, Yu Wang, Jing Cong, Xiaoke Wu

**Affiliations:** 1 Graduate School, Heilongjiang University of Chinese Medicine, Harbin, China; 2 Department of Obstetrics and Gynecology, First Affiliated Hospital of Zhejiang Chinese Medical University (Zhejiang Provincial Hospital of Traditional Chinese Medicine), Hangzhou, China; 3 The First Affiliated Hospital, Heilongjiang University of Chinese Medicine, Harbin, China

**Keywords:** polycystic ovary syndrome, metabolic score for insulin resistance (METS-IR), metabolism-related disorders, ovulatory dysfunction, insulin resistance

## Abstract

**Objective:**

This study aimed to evaluate the predictive value of the Metabolic Score for Insulin Resistance (Mets-IR) for metabolism-related disorders and its association with hormonal status and fertility outcomes in Chinese women with Polycystic Ovary Syndrome (PCOS).

**Methods:**

This secondary analysis included 957 women from the PCOSAct trial. Participants were stratified by Mets-IR quartiles. Linear regression analyzed correlations between Mets-IR and metabolic/hormonal parameters. ROC curves assessed Mets-IR’s predictive performance for metabolic disorders. Multivariable logistic regression evaluated associations with fertility outcomes.

**Results:**

Significant linear correlations were observed between Mets-IR and key metabolic parameters (e.g., HOMA-IR, TG, WHR) and hormonal parameters (PG, E2, FT, LH, FSH, LH/FSH ratio, SHBG, FAI, AMH) (all *P* < 0.05). Specifically, Mets-IR was negatively correlated with PG, E2, LH, SHBG and AMH, and positively correlated with FT and FAI. After adjusting for age and BMI, Mets-IR remained significantly negatively associated with LH, FSH, SHBG and AMH (all *P* < 0.01), while it showed a significant positive association with FAI (*P* < 0.001) and a significant negative association with TT (*P* < 0.05). Mets-IR exhibited superior independent predictive ability compared to BMI for key hormonal parameters, including SHBG (ΔR² = 17.2% vs 13.7%), FAI (ΔR² = 16.8% vs 14.9%), and AMH (ΔR² = 3.9% vs 2.8%). ROC analysis demonstrated high predictive performance of Mets-IR for IR (AUC = 0.814), MetS (AUC = 0.878), and NAFLD (AUC = 0.818). In predicting ovulation, Mets-IR demonstrated moderate predictive performance comparable to BMI (AUC = 0.606), with an optimal cut-off value of 23.46. Mets-IR was negatively associated with ovulation but not with other fertility outcomes (conception, clinical pregnancy, live birth, and pregnancy loss).

**Conclusion:**

In women with PCOS, Mets-IR demonstrates significant associations with both metabolic and hormonal parameters and exhibits superior predictive ability over BMI for key hormonal markers (SHBG, FAI, AMH). This index serves as an effective non-invasive predictor for IR, MetS, and NAFLD. For the assessment of ovulatory dysfunction, its predictive performance is comparable to that of BMI, yet it demonstrates no significant association with other fertility outcomes.

## Introduction

Polycystic Ovary Syndrome (PCOS) is one of the most common endocrine and metabolic disorders in women of reproductive age, with a global prevalence ranging from 6% to 20% (1). The disease exhibits highly heterogeneous clinical phenotypes. Beyond classic gynecological features (such as ovulatory dysfunction and polycystic ovarian morphology) and hyperandrogenism-related manifestations, PCOS patients often present with significant metabolic abnormalities (2). Insulin resistance (IR) lies at the core of these disturbances (3), which may further include metabolic syndrome (MetS), dyslipidemia, and non-alcoholic fatty liver disease (NAFLD) (4). These metabolic disorders not only directly impact reproductive health (e.g., reduced ovulation rate and increased infertility risk) but also significantly elevate the long-term risks of developing chronic conditions such as type 2 diabetes and cardiovascular diseases, thereby exerting multidimensional and long-term adverse effects on the overall health of PCOS patients (5).

Currently, the diagnosis of PCOS-related metabolic disorders faces several challenges. First, diagnostic outcomes are significantly influenced by variations in criteria, assessment methods, and population characteristics, making it difficult to establish a unified and precise evaluation system. On the other hand, existing indicators lack sufficient comprehensive predictive power for the multiple metabolic comorbidities in PCOS patients. Specifically, IR, as a central pathophysiological mechanism in PCOS, not only interacts synergistically with endocrine dysfunction (such as hyperandrogenemia) but also promotes metabolic diseases including obesity, hyperglycemia, and dyslipidemia through dysregulation of lipid and glucose metabolism (6). Although the hyperinsulinemic-euglycemic clamp technique remains the gold standard for quantifying IR, its invasive nature, technical complexity, and high cost limit its routine clinical use (7). The Homeostasis Model Assessment of Insulin Resistance (HOMA-IR), while widely adopted due to its non-invasiveness and simplicity, demonstrates considerable variability in accuracy across different populations (e.g., it shows significant bias in obese individuals or those with severely impaired β-cell function) (8, 9). The prevalence of MetS also varies widely among PCOS populations. Although some studies indicate a high overall rate of metabolic abnormalities in women with PCOS, the prevalence of MetS in Chinese PCOS patients assessed according to the International Diabetes Federation (IDF) criteria is relatively low (10), suggesting that ethnic-specific factors may influence diagnostic outcomes. The diagnosis of NAFLD also has limitations; liver biopsy, as the gold standard, is difficult to implement widely in clinical practice due to risks such as sampling error and invasive complications (11). Moreover, obesity (particularly abdominal obesity) and dyslipidemia are closely interrelated with IR and MetS, collectively contributing to the progression of PCOS and further complicating metabolic assessment (12). These diagnostic limitations highlight an urgent need to identify a comprehensive, non-invasive, and reliable biomarker for accurate prediction and dynamic monitoring of metabolic abnormalities in PCOS patients.

The Metabolic Score for Insulin Resistance (Mets-IR) is a recently proposed novel metabolic indicator. Its core advantage lies in integrating multidimensional metabolic information—including blood glucose (fasting glucose), lipids (triglycerides, high-density lipoprotein cholesterol), and body mass index (BMI)—providing a comprehensive reflection of metabolic status. Previous studies have demonstrated that Mets-IR exhibits good predictive value for IR in the general population and is significantly associated with the risks of MetS and cardiovascular diseases (13). However, its utility in Chinese PCOS populations has not been systematically investigated: on one hand, its comprehensive predictive ability for various metabolic diseases (such as IR, MetS, dyslipidemia, central obesity, and NAFLD) in PCOS patients remains unclear; on the other hand, the relationship between Mets-IR and fertility outcomes (including ovulation rate, conception rate, clinical pregnancy rate, pregnancy loss rate, and live birth rate) in PCOS patients remains unexplored.

Therefore, based on baseline data from the Acupuncture and Clomiphene Trial for Polycystic Ovary Syndrome (PCOSAct), this study aims to: (1) systematically evaluate the predictive efficacy of Mets-IR for metabolic disorders—including IR, MetS, dyslipidemia, central obesity, and NAFLD—in Chinese women with PCOS; (2) investigate the correlation between Mets-IR and hormonal parameters in PCOS patients; and (3) analyze the correlation between Mets-IR and fertility outcomes (ovulation, conception, clinical pregnancy, pregnancy loss, and live birth) in these patients. We hypothesize that Mets-IR can serve not only as an effective tool for identifying metabolic risk in PCOS patients but also provide a new reference for clinical individualized fertility management strategies, thereby offering theoretical support and practical guidance for integrated metabolic-reproductive management in PCOS.

## Materials and methods

### Participants

This secondary analysis utilized data from the PCOSAct, a large-scale, multicenter, randomized, double-blind, controlled clinical trial (ClinicalTrials.gov Identifier: NCT01573858; Chinese Clinical Trial Registry Number: ChiCTR-TRC-12002081). The study enrolled 1,000 infertile women diagnosed with PCOS according to the modified Rotterdam criteria (14), aged 20 to 40 years, across 27 hospitals in mainland China between 2012 and 2015. Both the trial protocol and primary outcomes have been previously published (15). Exclusion criteria comprised: endocrine comorbidities; recent (within the past 2 months) use of hormonal or herbal medications; perinatal status (≤6 weeks post-delivery/miscarriage); ongoing breastfeeding; missing metabolic data; and pregnancy loss with loss to follow-up.

### Anthropometric measurements

At baseline, all participants underwent a physical examination that included the assessment of age, height, weight, waist circumference (WC), hip circumference (HC), acanthosis nigricans score (AN), systolic blood pressure (SBP), and diastolic blood pressure (DBP). Body mass index (BMI = weight [kg]/height² [m²]) and waist-to-hip ratio (WHR = waist [cm]/hip [cm]) were calculated. Data regarding menstrual history were also collected, including age at menarche, average menstrual cycle length, average number of menstrual periods per year, and ovulatory cycles.

### Biochemical measurements

Fasting blood samples were collected at baseline following a 12-hour overnight fast. For regularly cycling women, samples were obtained on day 3 of the menstrual cycle, while women with amenorrhea provided samples on their enrollment day. All samples were analyzed at the core laboratory of Heilongjiang University of Chinese Medicine. All samples were analyzed at the core laboratory of Heilongjiang University of Chinese Medicine. A comprehensive biochemical analysis was performed, which included: Sex hormone parameters[Progesterone (PG), estradiol (E2), free testosterone (FT), total testosterone (TT), luteinizing hormone (LH), follicle-stimulating hormone (FSH), LH/FSH ratio, sex hormone-binding globulin (SHBG), and anti-Müllerian hormone (AMH)];Metabolic parameters[Fasting insulin (FINS), fasting plasma glucose (FPG), high-density lipoprotein (HDL), low-density lipoprotein (LDL), total cholesterol (TC), triglycerides (TG),TC/HDL ratio, TG/HDL ratio, ApoB/ApoA1 ratio, apolipoprotein A1 (ApoA1), apolipoprotein B (ApoB), lipoprotein(a) [Lp], lactate dehydrogenase (LDH), creatine kinase (CK), creatine kinase-MB isoenzyme (CK-MB), cystatin C (CysC),β2-microglobulin (B2-MG), and homocysteine (HCY)];Liver function parameters[Alanine aminotransferase (ALT), aspartate aminotransferase (AST), AST/ALT ratio, total bile acid (TBA), total bilirubin (TBIL), direct bilirubin (DBIL), and indirect bilirubin (IBIL)].Derived indices were calculated as follows: Free androgen index (FAI) = TT (nmol/L)/SHBG (nmol/L) × 100; Homeostasis model assessment of insulin resistance (HOMA-IR) = [FPG (mmol/L) × FINS (mIU/L)]/22.5 (16); atherogenic index (AI) = (TC–HDL)/HDL (17), atherogenic index of plasma (AIP) = log10 (TG/HDL) (18), lipid accumulation product (LAP) = (WC (cm) – 58) × TG (mmol/L) for females (19), visceral adiposity index (VAI) = {WC (cm)/[36.58 + (1.89 × BMI (kg/m²))]} × (TG (mmol/L)/0.81) × (1.52/HDL (mmol/L)) for females and triglyceride-glucose index (TyG=Ln[TG (mg/dL)×FPG (mg/dL)/2]) (20, 21).

In this study, a HOMA-IR cut-off value of 2.69 was adopted to define insulin resistance (22, 23). MetS was defined by presenting three or more of the following five items (14) (1): WC >88 cm; (2) SBP ≥130 mmHg and/or DBP ≥85 mmHg; (3) FPG level of 110–126 mg/dl (to convert to millimoles per liter, multiply by 0.0555); (4) TG level ≥150 mg/dl (to convert to millimoles per liter, multiply by 0.0113); and (5) HDL level <50 mg/dl (to convert to millimoles per liter, multiply by 0.0259)(4). Dyslipidemia was defined in accordance with the Chinese Guidelines on Prevention and Treatment of Dyslipidemia in Adults as follows: TC levels ≥ 6.22 mmol/L, TG levels ≥ 2.26 mmol/L, LDL-C levels ≥ 4.14 mmol/L, and HDL-C levels ≤ 1.04 mmol/L (24).Central obesity was defined as a waist-to-hip ratio (WHR) ≥ 0.85 (25).Nonalcoholic fatty liver Disease (NAFLD) was determined based on prior physical examination, without additional specific measurements conducted. The MetS-IR was calculated for each participant using the following formula (26):MetS-IR = Ln[(2 × FPG (mg/dL)) + TG (mg/dL)] × BMI (kg/m²)/Ln[HDL-C (mg/dL)].

### Pregnancy outcomes

Ovulation was defined as a serum PG level > 5 ng/mL during a cycle. Conception was defined as a serum hCG level > 10 IU/L, and all interventions were discontinued. Pregnancy was defined as an intrauterine pregnancy confirmed by transvaginal ultrasound, with fetal heart pulsations. Live birth was defined as the delivery of a viable infant at 20 weeks or later gestation. Pregnancy losses included miscarriages, fetal deaths, and stillbirths. According to the PCOSAct trial protocol, the core treatment period in this study is 16 weeks (equivalent to 4 standard menstrual cycles). If a participant experiences prolonged cycles following ovulation (e.g., a 5-week cycle), the treatment period may be extended up to 20 weeks to ensure the completion of 32 acupuncture sessions. In cases where pregnancy occurs during this period (i.e., during ART), specific follow-up commences upon confirmation of pregnancy and continues until either delivery or pregnancy termination. Key follow-up assessments include confirmation of fetal viability around the end of the first trimester (approximately 9 weeks of gestation), with continued monitoring until delivery and up to 42 days (6 weeks) postpartum.

### Statistical analysis

Data analysis was performed using SPSS Statistics version 26.0 (IBM SPSS Inc., Chicago, IL, USA). Continuous variables are presented as mean ± standard deviation, while categorical variables are expressed as frequencies and percentages. Linear trend tests were applied to compare differences in anthropometric measures, biochemical parameters, and the prevalence of metabolism-related disorders across Mets-IR quartiles. Linear regression was used to analyze the correlation between Mets-IR and key metabolic indicators. Receiver operating characteristic (ROC) curve analysis was conducted to evaluate the predictive performance of Mets-IR for metabolism-related diseases, and the AUC, optimal cutoff value, sensitivity, specificity, and Youden’s index were calculated. Multivariable logistic regression analysis was employed to estimate the odds ratios (OR) and 95% confidence intervals (CI) for the associations between Mets-IR (as the independent variable) and metabolism-related disorders as well as various fertility outcomes (as dependent variables). A *P*-value < 0.05 was considered statistically significant, and a *P*-trend < 0.05 indicated a statistically significant trend.

## Result

Of the 1000 women initially enrolled in the PCOSAct, 957 participants with complete data were included in this study. Based on their MetS-IR values, participants were stratified into four nearly equal quartiles (Q1–Q4): Q1: ≤ 22.36 (n = 240);Q2: 22.37–27.30 (n = 239);Q3: 27.31–31.71 (n = 240);Q4: ≥ 31.72 (n = 238).

### The Anthropometric, biochemical characteristics, incidence of metabolism-related diseases and fertility outcomes of the participants across the quartiles of MetS-IR

As shown in **Table 1**, all anthropometric parameters showed significant differences across the MetS-IR quartile groups (Q1 to Q4). Specifically, age, height, weight, BMI, HC, WC, WHR, AN, SBP, and DBP all increased significantly with ascending quartiles, reaching the highest values in the Q4 group (all *P* < 0.001, all *P*-trend < 0.001).

**Table 1:** The anthropometric, biochemical characteristics, incidence of metabolism-related diseases and fertility outcomes of the participants across the quartiles of MetS-IR.

Variables	Q 1 (N = 240)	Q 2 (N = 239)	Q 3(N = 240)	Q 4 (N = 238)	*P*-value	*P*-trend
Anthropometric measures						
Age(year)	27.37 ± 2.94	27.94 ± 3.23	27.86 ± 3.48	28.51 ± 3.43*^#^	0.002	<0.001
Height (cm)	160.37 ± 5.04	160.80 ± 4.95*	161.25 ± 5.27*^△^	162.38 ± 4.90*^△#^	<0.001	<0.001
Weight (kg)	50.26 ± 4.63	57.98 ± 4.90*	65.39 ± 5.78*^△^	78.45 ± 10.21*^△#^	<0.001	<0.001
BMI (kg/m^2^)	19.53 ± 1.43	22.42 ± 1.31*	25.11 ± 1.54*^△^	29.69 ± 3.17*^△#^	0.000	0.000
HC (cm)	90.57 ± 5.67	95.44 ± 5.28*	100.11 ± 5.31*^△^	107.35 ± 7.38*^△#^	<0.001	<0.001
WC (cm)	74.92 ± 7.21	81.14 ± 6.48*	87.42 ± 6.72*^△^	98.15 ± 9.49*^△#^	<0.001	<0.001
WHR	0.83 ± 0.07	0.85 ± 0.06*	0.87 ± 0.06*^△^	0.92 ± 0.07*^△#^	<0.001	<0.001
AN	1.10 ± 0.32	1.11 ± 0.37	1.18 ± 0.43	1.42 ± 0.65*^△#^	<0.001	<0.001
SBP (mmHg)	108.49 ± 10.19	111.7 ± 8.87*	112.93 ± 8.12*^△^	116.20 ± 8.40*^△#^	<0.001	<0.001
DBP (mmHg)	72.6 ± 8.10	73.88 ± 7.17*	75.53 ± 7.93*^△^	77.24 ± 7.33*^△#^	<0.001	<0.001
Menstrual history						
Age at menarche (years)	14.10 ± 1.51	13.60 ± 1.40^*^	13.72 ± 1.46^*^	13.40 ± 1.37^*^	<0.001	<0.001
Average menstrual cycle (days)	61.94 ± 24.92	66.98 ± 44.62	71.74 ± 43.94^*^	77.45 ± 51.32^*△^	<0.001	<0.001
Average number of menstrual periods per year.	6.47 ± 1.77	6.38 ± 2.14	6.12 ± 2.21	5.80 ± 2.02^*△^	0.002	0.001
Ovulatory cycle	1.80 ± 1.23	1.81 ± 1.27	1.53 ± 1.23^*△^	1.42 ± 1.25^*△^	<0.001	<0.001
Sex hormone profiles						
PG (nmol/L)	3.57 ± 8.02	2.38 ± 4.69^*^	2.20 ± 2.30*	2.21 ± 3.40*	0.008	0.004
E2 (pmol/L)	312.08 ± 343.96	284.89 ± 323.58	258.13 ± 364.74	226.45 ± 220.42^*△^	0.024	0.002
FT (pg/ml)	2.06 ± 0.85	2.24 ± 0.83^*^	2.35 ± 0.85^*^	2.49 ± 0.79^*△^	<0.001	<0.001
TT (nmol/L)	1.62 ± 0.63	1.71 ± 0.66	1.64 ± 0.66	1.70 ± 0.64	0.343	0.363
LH (mIU/mL)	12.81 ± 6.40	11.38 ± 6.35^*^	9.86 ± 5.53^*△^	7.97 ± 4.09^*△^	<0.001	<0.001
FSH (mIU/mL)	6.30 ± 1.62	6.22 ± 1.68	5.91 ± 1.81^*△^	5.93 ± 1.51^*^	0.018	0.003
LH/FSH ratio	2.12 ± 1.18	1.85 ± 0.96^*^	1.80 ± 1.46^*^	1.35 ± 0.67^*△#^	<0.001	<0.001
SHBG (nmol/L)	61.72 ± 32.24	47.15 ± 30.43^*^	34.19 ± 23.5^*△^	27.45 ± 22.29^*△#^	<0.001	<0.001
FAI	3.49 ± 2.62	5.02 ± 3.78^*^	6.39 ± 4.31^*△^	8.36 ± 5.14^*△#^	<0.001	<0.001
AMH (ng/mL)	13.74 ± 6.95	12.59 ± 6.06^*^	11.72 ± 6.23^*^	10.24 ± 5.70^*△#^	<0.001	<0.001
Metabolic parameters						
FINS (pmol/L)	55.37 ± 72.6	73.26 ± 50.59^*^	102.20 ± 77.27^*△^	152.66 ± 109.82^*△#^	<0.001	<0.001
FPG (mmol/L)	4.84 ± 0.83	4.96 ± 0.85^*^	5.08 ± 0.84^*^	5.32 ± 1.28^*△#^	<0.001	<0.001
HOMA-IR	1.79 ± 2.67	2.40 ± 1.99^*^	3.48 ± 3.27^*△^	5.41 ± 4.88^*△#^	<0.001	<0.001
HDL (mmol/L)	1.53 ± 0.34	1.34 ± 0.39^*^	1.16 ± 0.29^*△^	1.07 ± 0.28^*△#^	<0.001	<0.001
LDL (mmol/L)	2.72 ± 0.76	2.92 ± 0.94^*^	3.02 ± 0.81^*△^	3.21 ± 0.90^*△#^	<0.001	<0.001
TC (mmol/L)	4.54 ± 0.98	4.67 ± 1.19	4.74 ± 1.02^*^	5.00 ± 1.12^*△#^	<0.001	<0.001
TG (mmol/L)	0.96 ± 0.38	1.34 ± 0.65^*^	1.75 ± 0.89^*△^	2.22 ± 1.03^*△#^	<0.001	<0.001
TC/HDL	3.05 ± 0.60	3.59 ± 0.84^*^	4.23 ± 1.06^*△^	4.84 ± 1.17^*△#^	<0.001	<0.001
TG/HDL	0.67 ± 0.35	1.08 ± 0.61^*^	1.63 ± 1.00^*△^	2.27 ± 1.37^*△#^	<0.001	<0.001
ApoA1 (g/L)	1.63 ± 0.31	1.54 ± 0.33^*^	1.44 ± 0.30^*△^	1.42 ± 0.28^*△#^	<0.001	<0.001
ApoB (g/L)	0.74 ± 0.20	0.84 ± 0.26^*^	0.94 ± 0.20^*△^	1.07 ± 0.29^*△#^	<0.001	<0.001
ApoAB/ApoA1	0.46 ± 0.12	0.56 ± 0.16^*^	0.67 ± 0.19^*△^	0.77 ± 0.19^*△#^	<0.001	<0.001
LDH(U/L)	77.99 ± 37.92	85.99 ± 40.6	91.91 ± 45.59^*^	96.45 ± 53.14^*△^	<0.001	<0.001
CK(U/L)	51.08 ± 33.19	56.15 ± 33.6	55.89 ± 27.97	59.89 ± 31.17^*^	0.029	0.005
B2-MG(mg/L)	1.23 ± 0.39	1.30 ± 0.38^*^	1.32 ± 0.39	1.37 ± 0.42^*^	0.003	<0.001
CYSC(mg/L)	0.44 ± 0.15	0.45 ± 0.13	0.47 ± 0.15^*^	0.50 ± 0.16^*△#^	<0.001	<0.001
HCY(umol/L)	7.43 ± 4.60	8.23 ± 5.00	8.58 ± 4.92^*^	9.08 ± 4.69^*^	0.003	<0.001
AIP	0.03 ± 0.30	0.02 ± 0.30	0.07 ± 0.30	0.07 ± 0.29	0.164	0.040
LAP	25.78 ± 16.18	37.16 ± 27.12^*^	47.76 ± 29.33^*△^	62.67 ± 45.40^*△#^	<0.001	<0.001
VAI	1.28 ± 0.70	2.09 ± 1.20^*^	3.17 ± 1.96^*△^	4.52 ± 2.79^*△#^	<0.001	<0.001
AI	2.05 ± 0.60	2.59 ± 0.84^*^	3.23 ± 1.06^*△^	3.84 ± 1.17^*△#^	<0.001	<0.001
TyG	8.13 ± 0.44	8.46 ± 0.51^*^	8.74 ± 0.55^*△^	9.02 ± 0.58^*△#^	<0.001	<0.001
Liver function parameters						
ALT(U/L)	5.82 ± 3.60	8.2 ± 7.87^*^	9.27 ± 9.15^*△^	12.83 ± 10.71^*△#^	<0.001	<0.001
AST(U/L)	11.20 ± 4.65	12.54 ± 6.30^*^	12.70 ± 7.25^*^	15.88 ± 9.58^*△#^	<0.001	<0.001
AST/ALT	2.40 ± 1.34	1.97 ± 0.98^*^	1.75 ± 0.88^*△^	1.58 ± 0.83^*△#^	<0.001	<0.001
TBA(umol/L)	2.26 ± 3.44	2.38 ± 4.03	1.75 ± 2.01^△^	1.46 ± 1.47^*△^	<0.001	<0.001
TBIL(umol/L)	7.27 ± 3.67	6.6 ± 3.18^*^	5.93 ± 2.72^*△^	5.75 ± 2.78^*△^	0.002	<0.001
DBIL(umol/L)	2.69 ± 1.60	2.38 ± 1.30^*^	2.17 ± 1.29^*^	1.95 ± 1.38^*△^	<0.001	<0.001
IBIL(umol/L)	4.61 ± 2.28	4.27 ± 2.06	3.84 ± 1.81^*△^	3.86 ± 1.96^*△^	<0.001	<0.001
Incidence of metabolism-related disorders						
IR	24(6.00%)	70(17.40%)^*^	123(30.60%)^*△^	185(46.00%)^*△#^	<0.001	<0.001
Mets	2(1.00%)	13(6.50%)^*^	42(21.10%)^*△^	142(71.40%)^*△#^	<0.001	<0.001
Central obesity	82(14.60%)	116(20.60%)^*^	161(28.60%)^*△^	203(36.10%)^*△#^	<0.001	<0.001
Dyslipidemia	30(7.40%)	78(19.30%)^*^	120(29.70%)^*△^	176(43.60%)^*△#^	<0.001	<0.001
NAFLD	0(0.00%)	6(8.80%)^*^	14(20.60%)^*^	48(70.60%)^*△#^	<0.001	<0.001
Fertility outcomes						
Ovulation	195(26.60%)	194(26.50%)	181(24.70%)	163(22.20%)^*^	0.001	<0.001
Conception	84(28.00%)	79(26.30%)	85(28.30%)	52(17.30%)	0.039	0.119
Clinical pregnancy	62(30.10%)	58(28.20%)	52(25.20%)	34(16.50%)	0.227	0.100
Live birth	59(30.40%)	53(27.30%)	52(26.80%)	30(15.50%)	0.484	0.139
Pregnancy loss	24(23.50%)	25(24.50%)	33(32.40%)	20(19.60%)	0.436	0.111

Significant differences are denoted as follows: *P<0.05 vs. Q1 group; ^△^*P* < 0.05 vs. Q2 group; ^#^*P* < 0.05 vs. Q3 group.

Regarding menstrual history, age at menarche showed a significant decreasing trend with increasing quartiles (*P* < 0.001, *P*-trend < 0.001), with Q2, Q3, and Q4 groups all having lower values than the Q1 group. In contrast, the average menstrual cycle length exhibited an increasing trend (*P* < 0.001, *P*-trend < 0.001), while both the average number of menstrual periods per year and the number of ovulatory cycles showed significant decreasing trends (*P* < 0.005, *P*-trend < 0.005), with the Q4 group being significantly lower than the Q1 and Q2 groups.

In terms of sex hormone profiles, PG, E2, LH, FSH, LH/FSH ratio, SHBG, and AMH all exhibited decreasing trends (*P* < 0.005, *P*-trend < 0.005). Conversely, FT and FAI showed significant increasing trends (both *P* < 0.001, *P*-trend < 0.001), with values in the Q4 group being significantly higher than those in the first three quartile groups. No significant differences or trends were observed in TT across the quartiles (*P* = 0.343, *P*-trend = 0.363).

For metabolic parameters, FINS, FPG, HOMA-IR, LDL, TC, TG, TC/HDL ratio, TG/HDL ratio, ApoB, ApoB/ApoA1 ratio, LDH, CK, B2-MG, CysC, HCY, LAP, AI and TyG all demonstrated significant increasing trends with rising quartiles (all *P* < 0.05, *P*-trend < 0.05), and reached their highest values in the Q4 group. In contrast, HDL and ApoA1 showed significant decreasing trends (both *P* < 0.001, *P*-trend < 0.001).

With respect to liver function parameters, ALT and AST increased significantly with ascending quartiles (both *P* < 0.001, *P*-trend < 0.001). Meanwhile, the AST/ALT ratio, TBA, TBIL, DBIL, and IBIL all exhibited significant decreasing trends (all *P* < 0.005, *P*-trend < 0.001).

Regarding the incidence of metabolism-related diseases, the prevalence rates of IR, MetS, Central obesity, dyslipidemia, and NAFLD all increased significantly with increasing quartiles (all *P* < 0.001, *P*-trend < 0.001). The Q4 group had the highest prevalence rates, which were significantly higher than those in the other three quartile groups.

In terms of fertility outcomes, the ovulation rate showed a significant decreasing trend with ascending quartiles (*P* = 0.001, *P*-trend < 0.001), and the ovulation rate in the Q4 group was significantly lower than that in the Q1 and Q2 groups. However, no significant linear trends were observed in conception rate, clinical pregnancy rate, pregnancy loss rate, or live birth rate across the quartile groups (all *P* > 0.05, *P*-trend > 0.05).

BMI, body mass index; HC, hip circumference; WC, waist circumference; WHR, waist-to-hip ratio; AN, acanthosis nigricans score; SBP, systolic blood pressure; DBP, diastolic blood pressure; PG, progesterone; E2, estradiol; FT, free testosterone; TT, total testosterone; LH, luteinizing hormone; FSH, follicle-stimulating hormone; SHBG, sex hormone-binding globulin; FAI, free androgen index; AMH, anti-Müllerian hormone; FINS, fasting insulin; FPG,; HOMA-IR, homeostatic model assessment-insulin resistance; HDL, high-density lipoprotein; LDL, low-density lipoprotein; TC, total cholesterol; TG, triglycerides; ApoA1, apolipoprotein A1; ApoB, apolipoprotein B; LDH, lactate dehydrogenase; CK, creatine kinase; B2-MG,β2-microglobulin; CYSC, cystatin C; HCY, homocysteine; AIP, atherogenic index of plasma; LAP, lipid accumulation product; VAI, visceral adiposity index; AI, atherogenic index; TyG, triglyceride-glucose index; ALT, alanine aminotransferase; AST, aspartate aminotransferase; TBA, total bile acid; TBIL, total bilirubin; DBIL, direct bilirubin; IBIL, indirect bilirubin; IR, insulin resistance; MetS, metabolic syndrome; NAFLD, non-alcoholic fatty liver disease.

### Linear regression analysis of Mets-IR with key metabolic parameters

Linear regression analysis revealed significant linear correlations between Mets-IR and multiple metabolic parameters as well as hepatic enzyme indicators (all *P* < 0.01). After adjustment for age and BMI, Mets-IR remained significantly positively associated with WC, FPG, HOMA-IR, TG, ApoB/ApoA1 ratio, VAI, LAP, WHR, AST, ALT, and TyG index (all *P* < 0.05), while it was significantly negatively associated with HDL (*P* < 0.001). In contrast, no significant associations were observed between Mets-IR and HC, SBP, DBP, TC, or LDL after adjustment (all *P* > 0.05)(as shown in **Table 2**).

**Table 2:** Linear regression analysis of Mets-IR with key metabolic parameters.

Variables	Crude	*P*	Adjusted^a^	*P*	Adjusted^b^	*P*
	β; 95% CI		β; 95% CI		β; 95% CI	
WC (cm)	0.811(0.396-0.434)	<0.001	0.806(0.393-0.431)	<0.001	0.046(0.007-0.040)	0.005
HC (cm)	0.776(0.501-0.556)	<0.001	0.771(0.498-0.552)	<0.001	-0.005(-0.025-0.017)	0.729
SBP (mmHg)	0.306(0.154-0.230)	<0.001	0.295(0.147-0.223)	<0.001	0.013(-0.004-0.020)	0.192
DBP (mmHg)	0.239(0.133-0.225)	<0.001	0.234(0.129-0.221)	<0.001	0.014(-0.003-0.024)	0.129
FPG (mmol/L)	0.223(0.959-1.697)	<0.001	0.221(0.950-1.683)	<0.001	0.026(0.045-0.263)	0.006
HOMA-IR	0.403(0.554-0.742)	<0.001	0.400(0.549-0.736)	<0.001	0.078(0.096-0.156)	<0.001
TC (mmol/L)	0.173(0.590-1.262)	<0.001	0.164(0.543-1.213)	<0.001	-0.005(-0126-0.070)	0.575
TG (mmol/L)	0.533(3.808-3.772)	<0.001	0.525(3.031-3.726)	<0.001	0.245(1.504-1.645)	<0.001
HDL (mmol/L)	-0.440(-0.202to-0.156)	<0.001	-0.432(-0.199to-0.153)	<0.001	-0.199(-0.086to-0.075)	<0.001
LDL (mmol/L)	0.215(1.026-1.857)	<0.001	0.207(0.971-1.800)	<0.001	-0.003(-0.141-0.106)	0.781
ApoAB/ApoA1	0.568(14.937-17.965)	<0.001	0.562(14.748-17.8070	<0.001	0.175(4.583-5.555)	<0.001
VAI	0.568(1.369-1.650)	<0.001	0.561(1.348-1.632)	<0.001	0.278(0.720-0.759)	<0.001
LAP	0.405(0.059-0.080)	<0.001	0.397(0.072-0.028)	<0.001	0.179(0.028-0.033)	<0.001
WHR	0.472(33.353-42.316)	<0.001	0.468(33.019-41.938)	<0.001	0.033(1.009-4.241)	0.001
AST(U/L)	0.231(0.133-0.232)	<0.001	0.236(0.137-0.235)	<0.001	0.025(0.005-0.035)	0.008
ALT(U/L)	0.301(0.162-0.244)	<0.001	0.302(0.163-0.245)	<0.001	0.032(0.009-0.034)	<0.001
TyG	0.555(4.748-5.7504)	<0.001	0.548(4.677-5.684)	<0.001	0.224(1.992-2.243)	<0.001

a Adjusting for age.

b Adjusting for age and BMI.

### Linear regression analysis of Mets-IR with hormonal status

Linear regression analysis revealed significant linear correlations between Mets-IR and multiple hormonal parameters in the unadjusted model (all *P* < 0.01). After adjustment for age and BMI, Mets-IR remained significantly negatively associated with LH, FSH, SHBG and AMH (all *P* < 0.01), while it showed a significant positive association with FAI (*P* < 0.001) and a significant negative association with TT (*P* < 0.05). In contrast, after adjustment, no significant associations were observed between Mets-IR and PG, E2, FT, or the LH/FSH ratio (all *P* > 0.05) (as shown in **Table 3**).

**Table 3:** Linear regression analysis of Mets-IR with hormonal status.

Variables	Crude	*P*	Adjusted^a^	*P*	Adjusted^b^	*P*
	β; 95% CI		β; 95% CI		β; 95% CI	
PG (nmol/L)	-0.109 (-0.197to-0.052)	<0.001	-0.100 (-0.187to-0.042)	0.002	-0.015 (0.037-0.004)	0.108
E2 (pmol/L)	-0.110 (-0.003to-0.001)	<0.001	-0.109 (-0.003to-0.001)	<0.001	-0.002 (0.000-0.000)	0.865
FT (pg/ml)	0.197 (0.926-1.797)	<0.001	0.201 (0.959-1.823)	<0.001	0.009 (-0.068-0.187)	0.362
TT (nmol/L)	0.034 (-0.268-0.886)	0.294	0.043 (-0.188t0-0.962)	0.187	-0.024 (-0.377to-0.050)	0.011
LH (mIU/mL)	-0.302 (-0.358to-0.238)	<0.001	-0.293 (-0.349to-0.229)	<0.001	-0.027 (-0.045to-0.008)	0.005
FSH (mIU/mL)	-0.098 (-0.569to-0.122)	0.003	-0.090 (-0.541to-0.096)	0.005	-0.030 (-0.170to-0.430)	<0.001
LH/FSH ratio	-0.232 (-1.520to-0.878)	<0.001	-0.222 (-1.467to-0.825)	<0.001	-0.013 (-0.162-0.030)	0.178
SHBG (nmol/L)	-0.415 (-0.091to-0.069)	<0.001	-0.412 (-0.091to-0.068)	<0.001	-0.071 (-0.017to-0.010)	<0.001
FAI	0.410 (0.464-0.618)	<0.001	0.411 (0.467-0.620)	<0.001	0.049 (0.039-0.090)	<0.001
AMH (ng/mL)	-0.198 (-0.239to-0.124)	<0.001	-0.187 (-0.229to-0.114)	<0.001	-0.034 (-0.048to-0.015)	<0.001

a Adjusting for age.

b Adjusting for age and BMI.

### Comparison of the predictive value of Mets-IR and BMI for hormonal parameters

In Model 1 (including BMI only), Mets-IR demonstrated significant unadjusted associations with most hormonal parameters, showing the highest proportion of explained variance for the FAI, SHBG, and LH. (all P < 0.001).In Model 2 (including both BMI and Mets-IR), Mets-IR maintained significant independent associations with SHBG, FAI, AMH, FSH and TT. (P < 0.005). Notably, the associations between Mets-IR and PG, E2, FT, and the LH/FSH ratio were no longer significant in Model 2 (all P > 0.05) (as shown in **Supplementary Table S1**).

### Diagnostic performance of Mets-IR for metabolism-related disorders

In **Supplementary Figure S1**, subfigures A, B, C, D and E respectively display the ROC curves of Mets-IR as a predictor for IR, MetS, Dyslipidemia, Central obesity, and NAFLD. For the prediction of IR, the analysis showed that the AUC was 0.814 (**Supplementary Figure S1A**); For the prediction of MetS, the analysis showed that the AUC was 0.878 (**Supplementary Figure S1B**); For the prediction of Dyslipidemia, the analysis showed that the AUC was 0.773 (**Supplementary Figure S1C**); For the prediction of Central obesity, the analysis showed that the AUC was 0.742 (**Supplementary Figure S1D**); For the prediction of NAFLD, the analysis showed that the AUC was 0.818 (**Supplementary Figure S1E**).The complete comparative data are shown in **Table 4**.

**Table 4:** The predictive value of Mets-IR in detecting metabolism-related disorders in women with PCOS.

Variables	AUC (95%CI)	Cutoff value	Sensitivity	Specificity	You den index	*P*‐value
IR	0.814(0.787-0.841)	27.935	0.744	0.750	0.494	0.000
MetS	0.878(0.853-0.903)	30.275	0.814	0.812	0.626	0.000
Dyslipidemia	0.773(0.744-0.803)	28.445	0.668	0.763	0.431	0.000
Central obesity	0.742(0.710-0.773)	28.250	0.594	0.785	0.379	0.000
NAFLD	0.818(0.776-0.861)	31.085	0.809	0.752	0.561	0.000

### Logistic regression analysis of Mets-IR with metabolism-related disorders in women with PCOS

**Table 5** presents the associations between Mets-IR and metabolism-Related Disorders across progressively adjusted models. In the unadjusted Model 1, Mets-IR showed significant positive correlations with IR, MetS, Dyslipidemia, Central obesity, and NAFLD. After adjusting for age (Model 2), these associations remained significant. Following further adjustment for BMI (Model 3), Mets-IR still showed significant positive correlations with IR, MetS, Dyslipidemia, Central obesity, and NAFLD.

**Table 5:** Logistic regression analysis of Mets-IR with metabolism-related disorders in women with PCOS.

Variables	Model 1	*P*-value	Model 2	*P*-value	Model 3	*P*-value
	OR; 95% CI		OR; 95% CI		OR; 95% CI	
IR	1.268(1.227-1.311)	<0.001	1.275(1.232-1.319)	<0.001	1.349(1.227-1.484)	<0.001
MetS	1.347(1.289-1.407)	<0.001	1.346(1.288-1.406)	<0.001	2.425(2.089-2.815)	<0.001
Dyslipidemia	1.205(1.170-1.241)	<0.001	1.202(1.167-1.238)	<0.001	3.903(3.264-4.666)	<0.001
Central obesity	1.191(1.156-1.227)	<0.001	1.191(1.156-1.228)	<0.001	1.114(1.017-1.219)	0.002
NAFLD	1.197(1.146-1.249)	<0.001	1.195(1.144-1.247)	<0.001	1.268(1.109-1.098)	<0.001

Model 1: Non-adjusted.

Model 2: Adjusted for age.

Model 3: Adjusted for age, BMI.

### Predictive performance of Mets-IR and other metabolic parameters for MetS

In **Supplementary Figure S2** demonstrates the predictive performance of Mets-IR and other metabolic parameters for MetS. All indicators showed favorable predictive ability for MetS. Among them, Mets-IR exhibited the most outstanding overall predictive performance, with an AUC of 0.878. Complete comparative data is provided in **Table 6**.

**Table 6:** Mets-IR and other metabolic indicators predict AUC, cutoff value, sensitivity, and specificity prediction values for MetS.

Variables	AUC (95%CI)	Cutoff value	Sensitivity	Specificity	You den index	*P*‐value
Mets-IR	0.878(0.853-0.903)	30.275	0.814	0.812	0.626	0.000
Tyg	0.871(0.844-0.899)	8.905	0.814	0.829	0.643	0.000
TG/HDL	0.866(0.84-0.892)	1.475	0.834	0.793	0.627	0.000
TG	0.862(0.834-0.889)	1.715	0.839	0.803	0.642	0.000
TC/HDL	0.843(0.815-0.871)	4.135	0.834	0.732	0.566	0.000
ApoB/ApoA1	0.827(0.796-0.858)	0.635	0.849	0.699	0.548	0.000
WHR	0.776(0.741-0.811)	0.875	0.829	0.622	0.451	0.000
BMI	0.825(0.795-0.855)	24.88	0.814	0.721	0.535	0.000

### Logistic regression analysis of Mets-IR with ovulation and pregnancy outcomes in women with PCOS

**Table 7** presents the association between Mets-IR and fertility outcomes across progressively adjusted models. In the unadjusted Model 1, Mets-IR showed a significant negative association with ovulation. After further adjustment for treatment regimen and age in Model 2, this negative association remained statistically significant. However, the association was lost after further adjustment for BMI. In contrast, no significant association was observed between Mets-IR and final pregnancy outcomes.

**Table 7:** Logistic regression analysis of Mets-IR with ovulation and pregnancy outcomes in women with PCOS.

Variables	Model 1	*P*-value	Model 2	*P*-value	Model 3	*P*-value
	OR; 95% CI		OR; 95% CI		OR; 95% CI	
Ovulation	0.938(0.913-0.962)	<0.001	0.935(0.909-0.961)	<0.001	0.971(0.881-1.070)	0.552
Conception	0.982(0.956-1.008)	0.166	0.987(0.961-1.014)	0.339	0.944(0.861-1.035)	0.216
Clinical pregnancy	0.972(0.931-1.015)	0.199	0.973(0.932-1.016)	0.217	0.975(0.932-1.019)	0.258
Pregnancy loss	1.030(0.987-1.076)	0.171	1.026(0.982-1.072)	0.245	1.025(0.980-1.071)	0.280
Live birth	0.972(0.931-1.015)	0.196	0.978(0.935-1.022)	0.315	0.978(0.935-1.022)	0.315

Model 1: Non-adjusted.

Model 2: Adjusted for treatment, age.

Model 3:Adjusted for treatment, age, BMI.

### Predictive performance of Mets-IR compared with BMI for ovulation in PCOS

**Figure 1:**
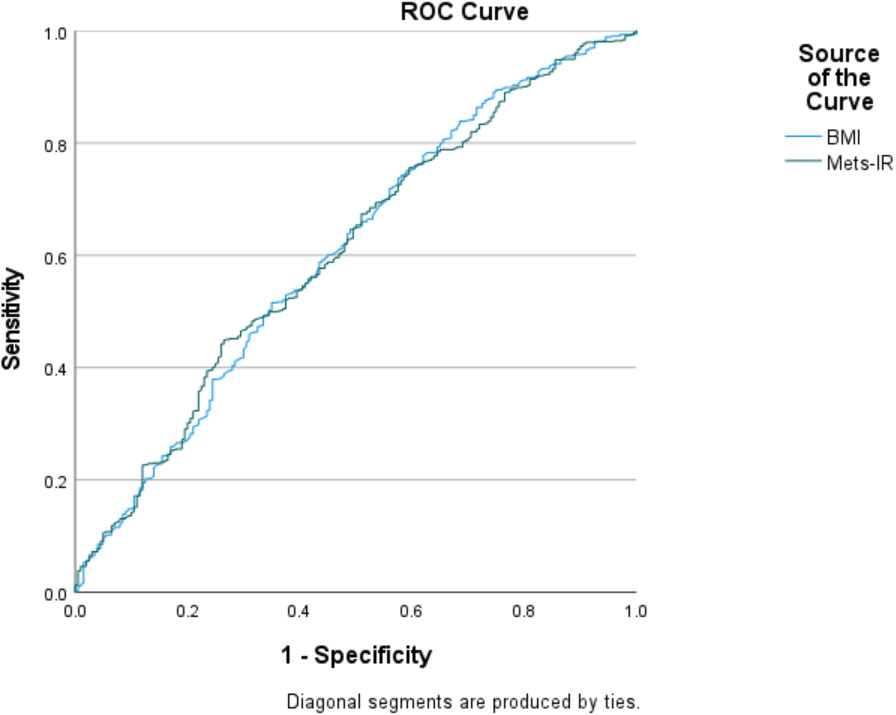
The results of ROC curve analysis regarding the predictability of Mets-IR and BMI for Ovulation.

**Figure 1** demonstrates the predictive performance of Mets-IR and BMI for ovulation. Both Mets-IR and BMI showed identical area under the ROC curve (AUC) values of 0.606, indicating moderate and comparable predictive ability for ovulatory dysfunction. Although the AUC values were identical, BMI demonstrated higher specificity while Mets-IR showed slightly higher sensitivity. Complete comparative data are presented in **Table 8**.

**Table 8:** Mets-IR and BMI predict AUC, cutoff value, sensitivity, and specificity prediction values for ovulation.

Variables	AUC (95%CI)	Cut off value	Sensitivity	Specificity	You den index	*P*‐value
Mets-IR	0.606(0.560-0.651)	23.460	0.516	0.650	0.166	0.000
BMI	0.606(0.561-0.650)	25.735	0.449	0.735	0.184	0.000

## Discussion

This study presents the first comprehensive evaluation of Mets-IR in Chinese women with PCOS. Our findings demonstrate that Mets-IR exhibits significant correlations not only with metabolic parameters but, more notably, with hormonal profiles and ovulatory function, confirming the specific metabolic-endocrine interplay in this disorder.

### Association between Mets-IR and hormonal status

A central finding of our study is the significant association between Mets-IR and several key hormonal parameters. Specifically, after adjusting for age and BMI, Mets-IR maintained statistically significant independent correlations with SHBG, FAI, and AMH. SHBG, a liver-synthesized carrier protein for sex hormones, has its expression directly regulated by insulin sensitivity (27). Hyperinsulinemia and subsequent insulin receptor dysfunction constitute the core mechanism suppressing hepatic SHBG synthesis and secretion. Specifically, insulin binding to its receptors on hepatocytes activates the insulin receptor substrate (IRS)/phosphatidylinositol 3-kinase (PI3K)/protein kinase B (Akt) signaling pathway (28). In insulin-resistant states, impaired signal transduction through this canonical pathway leads to attenuated phosphorylation-mediated suppression of the downstream transcription factor forkhead box O1 (FoxO1) (29, 30). Insufficiently suppressed FoxO1 persistently occupies the SHBG gene promoter region, thereby strongly inhibiting its transcriptional activity and ultimately resulting in significantly reduced circulating SHBG levels (31, 32). This mechanism explains the observed phenomenon in our study: as Mets-IR (reflecting the degree of insulin resistance) increases, SHBG levels demonstrate a significant independent decline. The consequent reduction in SHBG elevates free testosterone bioavailability, exacerbating clinical manifestations of hyperandrogenism (33). Thus, Mets-IR may serve as an important indirect driver of hyperandrogenemia in PCOS through its regulatory effect on SHBG expression.

Our study further revealed a significant independent positive correlation between Mets-IR and FAI, confirming the intrinsic link between metabolic disorders and hyperandrogenemia in PCOS (34).Mechanistically, the insulin-resistant state reflected by Mets-IR may elevate androgen levels through multiple synergistic pathways: on one hand, it upregulates the expression of insulin-like growth factor-1 receptors in ovarian theca cells, amplifying LH-mediated androgen synthesis (33); on the other hand, hyperinsulinemia can directly stimulate the adrenal cortex, particularly the zona reticularis, promoting the secretion of DHEA and its sulfate (DHEAS) (35). Furthermore, the chronic low-grade inflammation associated with insulin resistance may also contribute to androgen overproduction from both ovarian and adrenal sources via activation of signaling pathways such as NF-κB (36). In terms of clinical intervention, strategies aimed at improving insulin sensitivity have been demonstrated to effectively reduce FAI. Lifestyle interventions, such as dietary modification and regular physical activity, serve as foundational therapy and can significantly improve metabolic parameters and lower androgen levels (37). Pharmacologically, the insulin-sensitizing agent metformin, which activates the AMPK signaling pathway to ameliorate peripheral insulin resistance, has been shown to markedly reduce FAI in PCOS patients (38) Additionally, newer glucose-lowering agents such as GLP-1 receptor agonists have also been observed to lower FAI, alongside their benefits in weight reduction and metabolic improvement (39).

This study identified a specific independent correlation between Mets-IR and AMH, which deepens the understanding of the interaction between ovarian function and metabolic disturbances in PCOS. As a core biomarker of ovarian reserve, AMH levels are regulated by metabolic status. In the context of severe IR, the accompanying hyperandrogenic environment not only promotes excessive recruitment of primordial follicles (33) but may also directly disrupt the normal function of AMH signaling pathways, thereby weakening its physiological inhibitory effect on follicular development (40). In the long term, this regulatory imbalance may lead to a decline in the proportion of functional antral follicles, consequently resulting in reduced AMH levels (40). At the mechanistic level, in addition to the aforementioned impairment of signaling pathways, a hyperinsulinemic state can also induce excessive generation of reactive oxygen species (ROS) in granulosa cells and upregulate the expression of pro-inflammatory factors (e.g., TNF-α, IL-6), thereby directly inhibiting AMH synthesis and secretion (41). Moreover, the lipid metabolism abnormalities associated with high Mets-IR should not be overlooked. Elevated free fatty acids may impair mitochondrial function in granulosa cells via lipotoxic effects, triggering endoplasmic reticulum stress and apoptosis, which further contributes to decreased AMH levels (42, 43).

Previous studies have demonstrated that obese PCOS patients exhibit significantly lower LH levels and LH/FSH ratios compared to their non-obese counterparts (33), a finding consistent with our results. Elevated Mets-IR is frequently associated with obesity, which may suppress LH secretion through the following mechanisms. First, chronically elevated leptin secretion from adipose tissue induces central leptin resistance, thereby attenuating the pulsatile secretion of gonadotropin-releasing hormone (GnRH) (44); Second, enhanced aromatase activity in adipose tissue promotes the conversion of testosterone to E2, which subsequently inhibits pituitary LH release via negative feedback (45).

Furthermore, by comparing the predictive value of Mets-IR and BMI for hormonal parameters, this study found that both demonstrated similar capabilities in predicting SHBG and FAI. This finding is expected, given that BMI is a component of Mets-IR and obesity itself can influence hormone levels through the aforementioned mechanisms. However, it is noteworthy that even after adjusting for BMI, Mets-IR maintained independent associations with hormonal parameters. This suggests that the impact of Mets-IR on endocrine function is not entirely dependent on BMI, and that the glycemic and lipid parameters it integrates may provide metabolic information beyond what is captured by BMI alone. This observation aligns with findings reported by Duan et al (13). in the general population. Nevertheless, in the specific context of PCOS, the disease-specific implications of this association—such as its interaction with polycystic ovarian morphology—warrant further investigation.

### Association between Mets-IR and reproductive function in PCOS patients: a “metabolic indicator” of menstrual disturbance and ovulatory function

Menstrual disorders—including oligomenorrhea, irregular cycles, and even amenorrhea—represent a major clinical feature in women with PCOS. It is estimated that 85–90% of PCOS patients exhibit reduced ovulation frequency and prolonged menstrual cycles (46). A cross-sectional study demonstrated that women with intermenstrual intervals exceeding 35 days had significantly higher HOMA-IR levels compared to healthy controls, suggesting a positive correlation between the severity of oligomenorrhea and IR (47), a finding that aligns with our observation that PCOS patients with elevated Mets-IR tend to exhibit longer menstrual cycles and fewer annual menstrual episodes.

The findings of this study indicate a significant negative correlation between Mets-IR and ovulatory dysfunction in Chinese women with PCOS; however, no clear association was observed with downstream pregnancy outcomes, including conception, clinical pregnancy, and live birth. This discrepancy offers a new perspective for a deeper understanding of the “metabolic-reproductive interplay” in PCOS. In the unadjusted model, each unit increase in Mets-IR was associated with a 6.2% reduction in the risk of ovulation. This negative correlation remained statistically significant even after adjusting for treatment regimen and age. However, after additional adjustment for BMI, this association weakened and became non - significant, and the performance of Mets - IR in predicting ovulation was similar to that of BMI used alone. This is consistent with the complex pathophysiological mechanism of PCOS. In PCOS, ovulatory dysfunction is affected by multiple factors, including hormonal imbalances, insulin resistance of varying degrees, and obesity - related alterations (48).Moreover, obesity serves as a key driver of insulin resistance in women with PCOS, while insulin resistance further promotes ovarian androgen production and exacerbates endocrine disturbances, thereby perpetuating obesity and establishing a vicious cycle (49). Therefore, for overweight or obese PCOS patients, weight loss is considered a crucial intervention that can concurrently ameliorate metabolic abnormalities and restore reproductive function. This suggests that although Mets - IR is a valuable marker for identifying women with metabolic and ovulatory disorders, its ability to predict ovulation is not superior to the simpler BMI indicator in this PCOS population. This finding is of great significance for clinical practice, suggesting that BMI remains a highly relevant and easily accessible initial screening tool for evaluating ovulation potential. Future research can explore combining Mets - IR and BMI with other biomarkers to establish a more comprehensive predictive model for ovulation in PCOS.

In summary, from a mechanistic standpoint, elevated Mets-IR may suppress ovulation through the following pathways: on one hand, insulin resistance can potentiate the stimulatory effect of LH on ovarian theca cells—for instance, by upregulating LH receptor or IGF-1 receptor expression—thereby promoting androgen synthesis and exacerbating the hyperandrogenic state in PCOS patients (33, 50). This study also observed significantly lower SHBG levels in the high Mets-IR group, leading to an elevated FAI (34). On the other hand, PCOS patients often exhibit chronic low-grade inflammation and oxidative stress, wherein inflammatory cytokines (such as TNF-α and IL-6) and oxidative stress byproducts can directly impair the ovarian microenvironment and inhibit follicular maturation and the ovulation process (51).

### Predictive value of Mets-IR for IR, NAFLD, and MetS in PCOS patients

The findings of this study confirm that Mets-IR is closely associated with core metabolic comorbidities in PCOS, demonstrating significant clinical value particularly in the evaluation of IR, NAFLD, and MetS. As a central pathological mechanism in PCOS, IR concurrently drives dyslipidemia, abdominal obesity, NAFLD, and other metabolic disorders (34). This study observed that as Mets-IR quartiles increased, relevant glycolipid metabolic parameters and liver enzyme levels showed a significant trend of deterioration (13). Further analysis revealed a strong positive correlation between Mets-IR and HOMA-IR, along with very good predictive performance for IR. This supports the use of Mets-IR as a practical, non-invasive alternative indicator for assessing IR in PCOS patients, particularly in clinical settings with limited access to insulin testing, thereby effectively compensating for the limitations of traditional detection methods. Notably, Mets-IR also exhibited strong predictive capability for NAFLD. The prevalence of NAFLD in the PCOS population is as high as 43% (46), and it is a common comorbidity driven by both IR and dyslipidemia. Mechanistically, the insulin resistance represented by a high Mets-IR state promotes hepatic lipogenesis and fat accumulation, while concurrent dyslipidemia increases the transport of free fatty acids to the liver. These two processes act synergistically, ultimately leading to substantial triglyceride deposition within hepatocytes (11). By effectively capturing the complete pathophysiological cascade from IR to intrahepatic lipid deposition, Mets-IR serves as a useful screening tool for identifying PCOS patients at high risk of NAFLD. In a systematic comparison with commonly used metabolic indicators such as the TyG index, TG/HDL ratio, and BMI, this study further demonstrated that Mets-IR had the highest predictive accuracy for MetS, indicating its distinct advantage in the comprehensive assessment of metabolic abnormalities in PCOS patients.

In summary, as a convenient and efficient composite metabolic score, Mets-IR demonstrates multiple predictive utilities in the clinical management of PCOS. It not only effectively predicts decreased SHBG and elevated FAI—serving as an auxiliary tool for assessing hyperandrogenemia—but also shows an association with ovulatory dysfunction, although its specific applications require further validation in combination with other biomarkers. Furthermore, Mets-IR has demonstrated favorable predictive capability for metabolic disorders such as IR and NAFLD, supporting its use in clinical metabolic risk assessment.

### Strengths and limitations

The strengths of this paper lie in the following aspects: Firstly, the study benefits from a large sample size and comprehensive evaluation across multiple dimensions; Secondly, this is the first study to compare Mets-IR with conventional metabolic indicators in a PCOS population, demonstrating its superior predictive performance for MetS. Thirdly, Mets-IR relies solely on routinely measured clinical parameters, enhancing its practicality for widespread clinical use. However, the study has certain limitations: The cross-sectional design precludes the establishment of causal relationships between Mets-IR and metabolic or fertility outcomes. Moreover, the diagnosis of NAFLD was based on retrospective health records rather than gold-standard methods such as imaging or liver biopsy, which may lead to disease misclassification or underdiagnosis. Although the HOMA-IR cutoff is supported by existing literature, its discriminatory performance may vary across different PCOS phenotypes (e.g., obese vs. non-obese) and geographic populations (52) and its applicability in non-obese PCOS subgroups requires further validation. Furthermore, potential inter-assay variations could have influenced the results. Finally, the relatively low incidence of certain fertility outcomes, such as clinical pregnancy and live birth, limited the statistical power to detect significant associations in these areas.

## Conclusion and future work

In conclusion, this study establishes the Metabolic Score for Mets-IR as a highly effective, non-invasive, and integrative tool for the comprehensive assessment of Chinese women with PCOS. It demonstrates favorable overall predictive value for metabolism-related disorders, including Mets-IR and NAFLD. Furthermore, its significant associations with key hormonal parameters—specifically SHBG, FAI, and AMH—underscore its utility in capturing the critical metabolic-endocrine interplay that characterizes. While Mets-IR is clearly associated with ovulatory dysfunction and menstrual irregularities, its predictive performance for ovulation is comparable to, but not superior than BMI, highlighting the influence of adiposity in the pathophysiology of ovulatory disturbances. Notably, the attainment of final fertility outcomes, such as successful conception and live birth, is regulated by factors beyond metabolic indicators, including oocyte quality, endometrial receptivity, and embryonic developmental potential. For future work, longitudinal studies are warranted to track dynamic changes in Mets-IR over time and to establish its causal relationship with long-term metabolic and reproductive health outcomes in PCOS. Research should also focus on developing integrated prediction models that combine Mets-IR with other biomarkers (e.g., specific hormonal ratios, inflammatory markers, or ovarian sonographic features) to enhance the prediction of ovulation and other clinically relevant endpoints. Additionally, exploring the utility of Mets-IR for risk stratification and for guiding targeted interventions—such as lifestyle modifications or the use of insulin sensitizers in specific PCOS subphenotypes—represents a promising direction for personalized medicine. Finally, validating these findings in diverse ethnic populations and across different healthcare settings will be crucial to confirm the generalizability and clinical applicability of Mets-IR.

## Data Availability

The raw data supporting the conclusions of this article will be made available by the authors, without undue reservation.
