# Supplementary material for: The predictive value of the metabolic score for insulin resistance for metabolism-related disorders and fertility outcomes in Chinese women with polycystic ovary syndrome
Source: Front Endocrinol (Lausanne). 2025 Dec 5;16:1716287. doi: 10.3389/fendo.2025.1716287 (PMC12714639; doi:10.3389/fendo.2025.1716287)
Supplement: Supplementary file 1 [file DataSheet1.docx]

Supplementary Material

# Supplementary Data

# 1.1 Supplementary Figures and Tables

**Supplementary Table S1. Comparison of the Predictive Value of Mets-IR and BMI for Hormonal Parameters**

| Variables | **Model 1** | | | **Model 2** | | |
| --- | --- | --- | --- | --- | --- | --- |
|  | β; 95% CI | ****ΔR²**** | ***P*** | β; 95% CI | ****ΔR²**** | ***P*** |
| PG (nmol/L) | -0.096(-0.192to-0.039) | 0.009 | 0.003 | -0.109(-0.197to-0.052) | 0.012 | ＜0.001 |
| E2 (pmol/L) | -.0113(-13.246ti-3.746) | 0.013 | ＜0.001 | -0.110(-0.003to-0.001) | 0.012 | ＜0.001 |
| FT (pg/ml) | 0.198(0.027-0.052) | 0.039 | ＜0.001 | 0.197(0.926-1.797) | 0.039 | ＜0.001 |
| TT (nmol/L) | 0.062(0.000-0.019) | 0.004 | 0.054 | 0.034(-0.268-0.886) | 0.001 | 0.294 |
| LH (mIU/mL) | -0.287(-0.485to-0.314) | 0.082 | ＜0.001 | -0.302(-0.358to-0.238) | 0.091 | ＜0.001 |
| FSH (mIU/mL) | -0.069(-0.052to-0.002) | 0.005 | 0.034 | -0.098(-0.569to-0.122) | 0.010 | 0.003 |
| LH/FSH ratio | -0.227(-0.077to-0.044) | 0.051 | ＜0.001 | -0.232(-1.520to-0.878) | 0.054 | ＜0.001 |
| SHBG (nmol/L) | -0.370(-3.062to-2.214) | 0.137 | ＜0.001 | -0.415(-0.091to-0.069) | 0.172 | ＜0.001 |
| FAI | 0.368(0.341-0.464) | 0.149 | ＜0.001 | 0.410(0.464-0.618) | 0.168 | ＜0.001 |
| AMH (ng/mL) | -0.169(-0.347to-0.159) | 0.028 | ＜0.001 | -0.198(-0.239to-0.124) | 0.039 | ＜0.001 |

**Model 1：**Predictors: BMI；**Model 2：**Predictors: Mets-IR.

## 1.2 Supplementary Figures

**
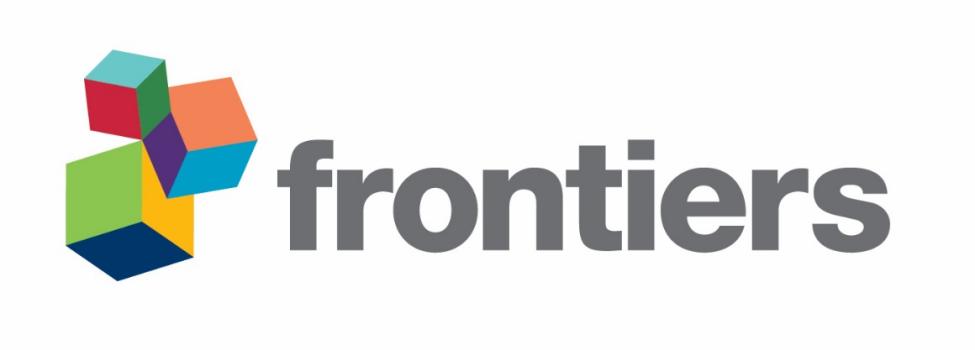
**

**Supplementary Figure S1.**

**Predictive Performance of Mets-IR for Metabolism-Related Disorders**


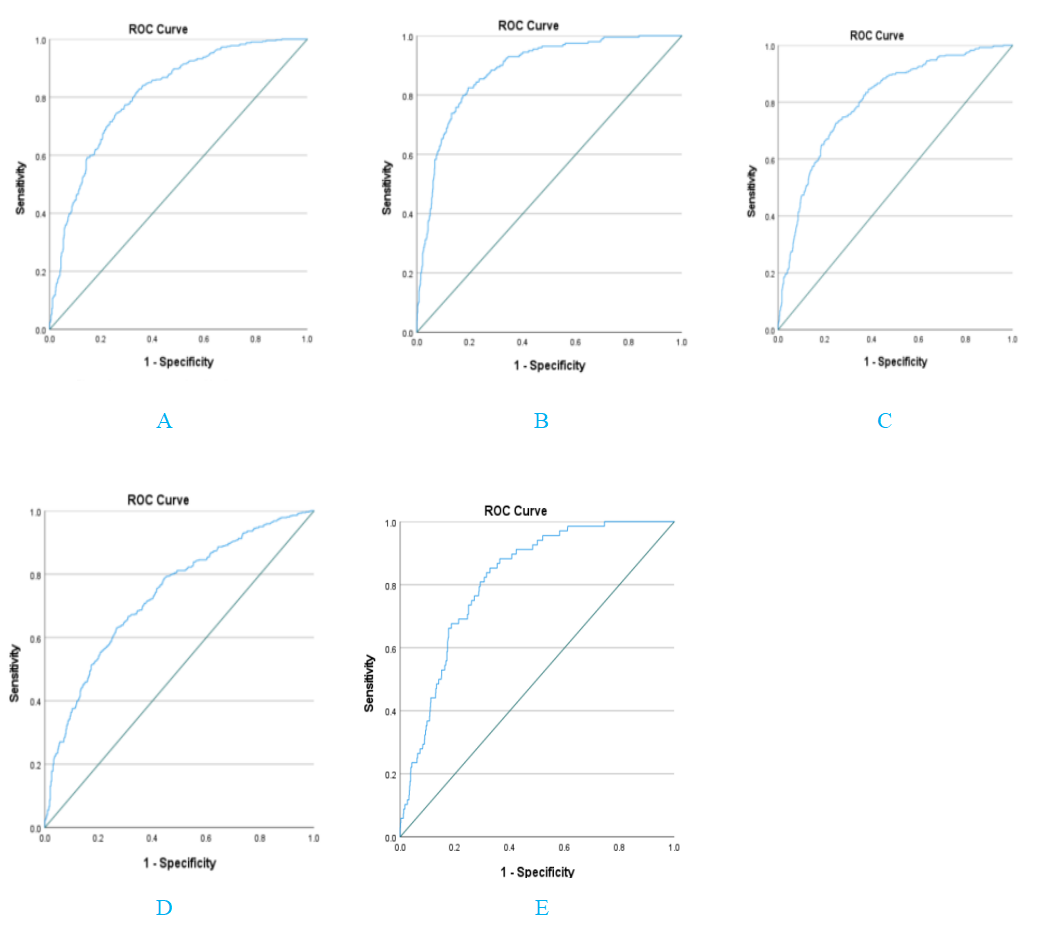


**Supplementary Figure S1. The results of ROC curve analysis regarding the predictability of Mets-IR in IR (Figure S1.A), MetS (Figure S1.B), Dyslipidemia(Figure S1.C), Central obesity(Figure S1.D), and NAFLD (Figure S1.E).**

**Supplementary Figure S2.**

**Predictive Performance of Mets-IR and other metabolic parameters for MetS**


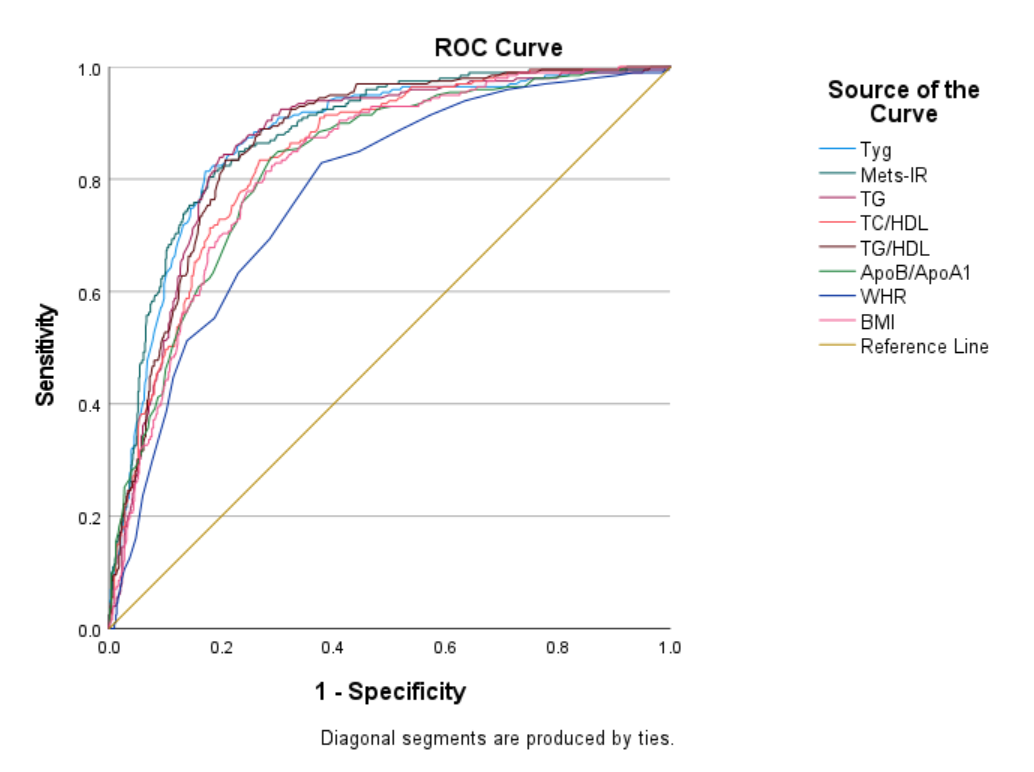


**Supplementary Figure S2. The results of ROC curve analysis regarding the predictability of Mets-IR and other metabolic parameters for MetS.**
